# Successful Management of Patients with Co-existent Graves’ Disease and Autoimmune Hepatitis

**DOI:** 10.7759/cureus.4647

**Published:** 2019-05-11

**Authors:** Sanah Rana, Zoubair Ahmed, Reena Salgia, Arti Bhan

**Affiliations:** 1 Internal Medicine, Riverview Medical Center, Red Bank, USA; 2 Internal Medicine, Raritan Bay Medical Center, Perth Amboy, USA; 3 Gastroenterology and Hepatology, Henry Ford Health System, Detroit, USA; 4 Endocrinology, Diabetes, and Metabolism, Henry Ford Health System, Detroit, USA

**Keywords:** graves' disease, autoimmune hepatitis, hyperthyroidism, elevated liver enzymes

## Abstract

Graves’ disease may lead to hepatic dysfunction. This is due to the direct effect of increased circulation of thyroid hormones. Graves’ disease is associated with other autoimmune diseases, including autoimmune hepatitis.

We report four cases of a rare occurrence of both Graves’ disease and autoimmune hepatitis. Two female patients underwent radioactive iodine ablation for Graves’ disease. Both patients were diagnosed with autoimmune hepatitis with liver biopsy after liver enzymes worsened despite stable thyroid function. Both patients received steroid immunosuppression therapy for autoimmune hepatitis. The first patient improved with return of thyroid function and liver enzymes to normal whereas the second patient’s liver disease progressed despite treatment and she eventually required liver transplant. A female patient with concomitantly diagnosed Graves’ disease and autoimmune hepatitis was initially treated with steroids and anti-thyroid medication. She then underwent radioactive iodine ablation but ultimately required liver transplant. Another female patient received treatment with immunosuppression and anti-thyroid therapy. She eventually underwent radioactive iodine ablation with normalization of thyroid function and liver profile.

This case series illustrates the diagnostic challenge to determine the cause of elevated liver enzymes in patients presenting with both Graves' disease and autoimmune hepatitis. A brief review of the literature on its clinical presentation and diagnosis is discussed.

## Introduction

Graves’ disease is an autoimmune disorder characterized by the presence of autoantibodies against thyroid-stimulating hormone (TSH) receptor [1]. Hyperthyroidism from Graves’ disease causes increased hepatocyte oxygen demand without increase in hepatic blood flow which can result in elevated liver enzymes [2,3]. Autoimmune hepatitis also presents with elevated liver enzymes, as well as gammaglobulinemia and unique histological features. These histological features include interface hepatitis and plasma cell infiltration [4]. For patients presenting with both Graves’ disease and hepatitis, the diagnostic challenge is to determine the cause of elevated liver enzymes. We report four patients diagnosed with both autoimmune hepatitis and Graves’ disease.

## Case presentation

The laboratory values of the four patients presented are shown in Table 1.

**Table 1:** Patient liver profile and thyroid studies at the time of diagnosis. ALT: Alanine aminotransferase; AST: Aspartate aminotransferase; TSH: Thyroid-stimulating hormone; T4: Levothyroxine *Initial liver profile at the time of diagnosis of autoimmune hepatitis. ^†^Initial thyroid profile at the time of diagnosis of Graves’ disease.

	ALT*	AST*	TSH^†^	Free T4^†^
Case 1	159	62	0.01	2.73
Case 2	127	108	0.09	2.03
Case 3	136	169	< 0.01	2.50
Case 4	606	1665	< 0.01	3.67
Reference range	< 78 IU/L	< 35 IU/L	0.45-5.33 uIU/mL	0.61-1.44 ng/dL

Case 1

A 15-year-old female presented with heat intolerance and palpitations. Laboratory tests revealed low TSH to 0.01 uIU/mL (reference range: 0.45-5.33 uIU/mL), elevated free T4 to 2.73 ng/dL (reference range: 0.61-1.44 ng/dL), and elevated thyroid-stimulating immunoglobulin to 422 U/mL (reference range: < 60 U/mL). Radioactive iodine uptake and scan showed diffusely increased homogenous uptake to 69% consistent with Graves’ disease. The patient was diagnosed with hyperthyroidism secondary to Graves’ disease and started on therapy with methimazole. Further laboratory testing revealed elevated levels of liver enzymes with aspartate aminotransferase (AST) of 62 IU/L (reference range: < 35 IU/L) and alanine aminotransferase (ALT) of 159 IU/L (reference range: < 72 IU/L) with normal bilirubin levels. Preexisting liver function tests were not available. Liver enzymes repeated after six weeks showed AST 189 IU/L and ALT 325 IU/L. Methimazole was discontinued. As a result, the patient underwent radioactive iodine ablation. She was later started on levothyroxine for post-ablative hypothyroidism. Liver enzymes continued to trend upward after eight weeks despite stable thyroid function. Laboratory workup revealed negative anti-nuclear antibody (ANA), anti-smooth muscle antibody, and hepatitis serologies. The decision was made to perform a liver biopsy given unclear cause of transaminitis. Liver biopsy showed portal fibrosis and interface hepatitis consistent with autoimmune hepatitis. Accordingly, oral steroid therapy was started for treatment of autoimmune hepatitis. After one month of steroid therapy, the liver enzymes normalized.

Case 2

A 21-year-old female presented with fatigue and palpitations. She was diagnosed with Graves’ disease after laboratory tests revealed low TSH to 0.09 uIU/mL, elevated free T4 to 2.03 mg/dL, and elevated thyroid-stimulating immunoglobulin to 89 U/mL. Liver profile was also obtained which showed AST 108 IU/L, ALT 127 IU/L, normal bilirubin, and normal alkaline phosphatase. Radioactive iodine uptake and scan revealed diffusely increased homogenous uptake consistent with Graves’ disease. Patient’s physician was hesitant to use anti-thyroid drugs for fear of worsening liver enzymes. Initial liver enzyme elevation was attributed to the hyperthyroidism itself. She then underwent radioactive iodine ablation for treatment of Graves’ disease. Afterwards, she was initiated on levothyroxine for post-ablation hypothyroidism. Her palpitations resolved and thyroid function normalized.

However, the patient continued to experience fatigue. Her liver enzymes were repeated after eight weeks and were notable for AST 111 IU/L and ALT 191 IU/L. Further workup revealed positive liver-kidney microsomal type 1 antibody to 320 U (reference range: < 20 U) with negative ANA and anti-smooth muscle antibody. A diagnosis of autoimmune hepatitis was confirmed after liver biopsy revealed bridging necrosis. The hepatitis progressed despite treatment with immunosuppression and she underwent a successful liver transplant two years later.

Case 3

A 39-year-old female presented with fatigue and worsening jaundice for three months. Laboratory tests were notable for low TSH to <0.01 uIU/mL, elevated AST to 136 IU/L, elevated ALT to 169 IU/L, elevated alkaline phosphatase to 466 IU/L (reference range: 44-147 IU/L), and elevated total bilirubin to 8 mg/dL (reference range: 0.1-1.2 mg/dL). Further workup revealed increased free T4 to 2.5 ng/dL and positive thyroid-stimulating immunoglobulin. Laboratory testing for ANA, anti-smooth muscle antibody, and liver-kidney microsomal type 1 antibody was negative but liver biopsy was consistent with autoimmune hepatitis. Radioactive iodine uptake and scan showed diffusely increased homogenous uptake consistent with Graves’ disease.

The patient was concurrently diagnosed with Graves’ disease and autoimmune hepatitis. She was started on prednisone and methimazole. The decision was made to definitively treat Graves’ disease with radioactive iodine ablation. After treatment, the patient went into remission of both Graves’ disease and autoimmune hepatitis. Her thyroid and liver tests remained stable until a few years later when she developed recurrence of autoimmune hepatitis. She eventually required a liver transplant at the age of 41.

Case 4

A 38-year-old female presented with marked jaundice and malaise. Laboratory tests were notable for elevated AST to 1665 IU/L, elevated ALT to 606 IU/L, elevated alkaline phosphatase to 123 IU/L, and elevated serum total bilirubin to 19.5 mg/dL. Further laboratory workup revealed positive anti-nuclear antibody (titer 1:5120) and positive anti-smooth muscle antibody titer. Liver biopsy revealed significant hepatic necrosis and bridging fibrosis, confirming the diagnosis of autoimmune hepatitis. The patient was started on immunosuppression. Her liver function eventually normalized and remained stable.

Fifteen years later, the patient developed symptoms of heat intolerance, tremors, and palpitations. Laboratory workup revealed low TSH to < 0.01 uIU/mL, elevated total T3 to 0.9 ng/dL, elevated free T4 to 3.67 ng/dL, and elevated serum thyroid-stimulating immunoglobulin to 281 U/mL. Radioactive iodine uptake and scan revealed diffusely increased homogenous uptake of 43% consistent with Graves’ disease. Her immunosuppression was continued for autoimmune hepatitis and she was initiated on methimazole for Graves’ disease. The patient’s clinical course was complicated by acute liver injury attributed to non-adherence with immunosuppression; however, hepatotoxicity from methimazole could not be ruled out. She received pulse dose steroids with normalization of liver enzymes. After initially refusing, she finally agreed to undergo radioactive iodine ablation six months later. Her liver enzymes remained stable and thyroid function returned to normal.

## Discussion

Hypothyroidism is commonly observed with other autoimmune disorders, including autoimmune hepatitis [5], but Graves’ disease in association with autoimmune hepatitis has not been frequently described in the literature [6,7]. Although the exact mechanism is unclear, autoimmune hepatitis and Graves’ disease are both considered to be immune mediated [1,4]. One retrospective study of patients with autoimmune hepatitis and concurrent autoimmune disease found Graves’ disease in only 1.8% of patients [7]. The prevalence of Graves’ disease in autoimmune hepatitis has been reported at 6% [8]. Notably, a recent study found that up to 10% of patients with Graves’ disease have a coexisting autoimmune disorder [5].

The function of the liver and the thyroid are closely intertwined. Thyroid hormones (T3 and T4) are essential for regulation of hepatic function, while the liver is involved in metabolism of thyroid hormones [2]. In vitro studies in animals demonstrated that excess T3 induces hepatocyte apoptosis and causes liver dysfunction [9]. Given that thyroid hormones are glucuronidated and sulfated in the liver, excess thyroid hormone can cause hepatocyte injury. Hepatic dysfunction in hyperthyroidism can be caused by the hyperthyroidism itself, heart failure resulting in hepatic venous congestion, or associated liver disease [2]. Liver profile abnormality from hyperthyroidism shows a hepatic pattern with elevations of serum ALT and AST. A cholestatic pattern with elevated alkaline phosphatase and bilirubin may also be seen in hyperthyroidism [2,10].

Since elevated liver enzymes are seen in patients with Graves’ disease, a high index of suspicion is required to diagnose concomitant autoimmune hepatitis. Liver histology and measurement of autoantibodies aid in the diagnosis of autoimmune hepatitis in patients with elevated liver enzymes [11]. The diagnosis of autoimmune hepatitis may be delayed in cases where the initial liver enzyme abnormalities are attributed to hyperthyroidism. Patients with Graves’ disease will have improvement of liver enzymes with treatment of hyperthyroidism [2]. Evaluation for an alternative cause of elevated liver enzymes should be initiated if liver profile worsens despite stable thyroid function. Viral hepatitis and primary biliary cholangitis are other possible causes of liver disease and should be investigated [11]. Improvement of liver enzymes is typically seen six to twelve weeks after initiation of treatment for autoimmune hepatitis [2].

Physicians may be hesitant to use anti-thyroid drug therapy given their hepatic side effect profile. However, it has been suggested that anti-thyroid drug therapy may be safely used in the presence of elevated liver enzymes and could prove beneficial. The presence of elevated liver enzymes is not a contraindication to use of anti-thyroid drugs [12,13]. Among anti-thyroid drugs, methimazole is the preferred agent in the treatment of hyperthyroidism as it is associated with less severe hepatic toxicity [14,15]. Methimazole-induced hepatotoxicity is frequently cholestatic whereas propylthiouracil use has been associated with hepatocellular injury, including fulminant hepatic failure [15,16]. As such, propylthiouracil should be used cautiously and is only preferred in the first trimester of pregnancy due to risk of methimazole-induced embryopathy [17]. In most patients, the liver profile will return to normal within three months after discontinuation of methimazole [10]. In addition, radioactive iodine ablation is the preferred modality for definitive treatment of Graves’ disease in patients with concomitant liver disease [18].

## Conclusions

In conclusion, liver enzyme elevation in Graves’ disease may be due to hyperthyroidism itself, anti-thyroid medication, or concomitant liver disease. For patients presenting with both Graves’ disease and hepatitis, the diagnostic challenge is to determine the cause of elevated liver enzymes. It is important to consider autoimmune hepatitis as a potential cause of liver dysfunction so appropriate therapy can be promptly initiated.

## References

[1] Smith TJ, Hegedüs L (2016). Graves’ disease. N Engl J Med.

[2] Khemichian S, Fong TL (2011). Hepatic dysfunction in hyperthyroidism. Gastroenterol Hepatol (NY).

[3] Shimizu Y (2008). Liver in systemic disease. World J Gastroenterol.

[4] Krawitt EL (2006). Autoimmune hepatitis. N Engl J Med.

[5] Boelaert K, Newby PR, Simmonds MJ (2010). Prevalence and relative risk of other autoimmune diseases in subjects with autoimmune thyroid disease. Am J Med.

[6] Sato I, Tsunekawa T, Shinohara Y, Nishio Y, Shimizu Y, Suzuki Y, Yoshioka S (2011). A case of autoimmune hepatitis with Graves’ disease treated with propylthiouracil. Nagoya J Med Sci.

[7] Teufel A, Weinmann A, Kahaly GJ (2010). Concurrent autoimmune diseases in patients with autoimmune hepatitis. J Clin Gastroenterol.

[8] Malik R, Hodgson H (2002). The relationship between the thyroid and the liver. QJM.

[9] Upadhyay G, Singh R, Kumar A, Kumar S, Kapoor A, Godbole MM (2004). Severe hyperthyroidism induces mitochondria-mediated apoptosis in rat liver. Hepatology.

[10] Lin TY, Shekar AO, Li N, Yeh MW, Saab S, Wilson M, Leung AM (2017). Incidence of abnormal liver biochemical tests in hyperthyroidism. Clin Endocrinol (Oxf).

[11] Jhee JH, Kim HJ, Kang W, Kim S, Kim DY (2015). A case of autoimmune hepatitis combined with Graves’ disease. Korean J Gastroenterol.

[12] Liaw YF, Huang MJ, Fan KD, Li KL, Wu SS, Chen TJ (1993). Hepatic injury during propylthiouracil therapy in patients with hyperthyroidism: a cohort study. Ann Intern Med.

[13] Niculescu DA, Dusceac R, Galoiu SA, Capatina CA, Poiana C (2016). Serial changes of liver function tests before and during methimazole treatment in thyrotoxic patients. Endocr Pract.

[14] Burch HB, Cooper DS (2015). Management of Graves’ disease: a review. JAMA.

[15] Nakamura H, Noh JY, Itoh K, Fukata S, Miyauchi A, Hamada N (2007). Comparison of methimazole and propylthiouracil in patients with hyperthyroidism caused by Graves’ disease. J Clin Endocrinol Metab.

[16] Ruiz JK, Rossi GV, Vallejos HA, Brenet RW, Lopez IB, Escribano AA (2003). Fulminant hepatic failure associated with propylthiouracil. Ann Pharmacother.

[17] Azizi F, Amouzegar A (2011). Management of hyperthyroidism during pregnancy and lactation. Eur J Endocrinol.

[18] Ross D, Burch H, Cooper D (2016). 2016 American Thyroid Association guidelines for diagnosis and management of hyperthyroidism and other causes of thyrotoxicosis. Thyroid.

